# Characterizing Ultra-Processed Foods by Energy Density, Nutrient Density, and Cost

**DOI:** 10.3389/fnut.2019.00070

**Published:** 2019-05-28

**Authors:** Shilpi Gupta, Terry Hawk, Anju Aggarwal, Adam Drewnowski

**Affiliations:** Department of Epidemiology, Center for Public Health Nutrition, University of Washington, Seattle, WA, United States

**Keywords:** NOVA classification, energy density, NRF_9.3_, monetary cost, ultra-processed foods, unprocessed foods, food frequency questionnaire (FFQ), food-based guidelines

## Abstract

**Background:** The NOVA food classification scheme divides foods into ultra-processed, processed, unprocessed, and culinary ingredients. Ultra-processed foods contribute >60% of energy to diets in the US.

**Objective:** To characterize ultra-processed foods by energy density, nutrient density, and monetary cost.

**Methods:** The 384 component foods of Fred Hutch (FHCRC) food frequency questionnaire (FFQ), were assigned to 4 NOVA categories and to 7 USDA MyPyramid food groups. Energy density was kcal/g. Nutrient density was measured using the Nutrient Rich Food index NRF_9.3_. Food prices were collected in local supermarkets from 2004 to 2016. Analyses examined time trends in food prices by NOVA category and by USDA food group.

**Results:** The ultra-processed classification captured mostly grains (91%), fats and sweets (73%), dairy (71%), and beans, nuts and seeds (70%), but only 36% of meat, poultry and fish, 26% of vegetables, and 20% of fruit. Compared to unprocessed foods, ultra-processed foods had lower nutrient density (NRF_9.3_ per 100 kcal: 21.2 vs. 108.5),higher energy density (mean (SD): 2.2 vs. 1.10 in kcal/g), and lower per calorie cost (0.55 vs. 1.45 in $/100 kcal). Ultra-processed foods did not increase in price as much as unprocessed foods over the 12 year period.

**Conclusion:** Ultra-processed foods tend to be energy-dense, low-cost, and nutrient-poor. Low energy cost could be one mechanism linking ultra-processed foods with negative health outcomes. Food-based Dietary Guidelines may need to address food processing in relation to economic aspects of food choice.

## Introduction

The NOVA food classification (1) has sought to establish food processing as the primary index of food quality. The four classes of foods were ultra-processed, processed, and unprocessed, as well as culinary ingredients (fat, sugar, salt) (2, 3). The definition of ultra-processed foods has varied over the years (1) and has not always been consistent (4–8). Ultra-processed foods were initially defined as industrial formulations with fats, sugars, and salt added during preparation, alongside other substances not used in normal cooking. Unprocessed foods were defined as those that were either fresh or that had gone through minimal processing (drying, freezing, pasteurization, or fermentation) mainly to make them safer, accessible and palatable (1). Most studies have contrasted the health impact of industrially engineered multi-ingredient ultra-processed foods with fresh or frozen vegetables and fruit and with unprocessed meat, poultry, and fish.

The level of food processing rather than the foods' nutrient content has thus been suggested as a potential framework for food and nutrition policy (9, 10). Ultra-processed foods, now linked to metabolic syndrome (11, 12), cancer (13), and all-cause mortality (14) are reported to pose a significant threat to human health (2, 15). Analyses of the nationally representative National Health and Nutrition Examination Surveys (NHANES) 2009–2010 suggest that ultra-processed foods accounted for 57.9% of dietary energy and almost 90% of added sugars in diets of US adults (16, 17). Purchase data for >1.2 million products from the 2000–2012 Homescan panel suggested that more than three-fourths of food energy purchased by US households came from highly processed foods and beverages (61%) (17). Similar figures were obtained for Canada (62%) (18) and the UK (63%) (19).

Most of the existing literature on ultra-processed foods, diet quality, and health outcomes has been based on examination of household food purchases (17) or individual diets (16). At this point little is known about the monetary cost of foods by NOVA category (20–22).

This study examined foods assigned into NOVA categories or USDA food groups by energy density, nutrient density, and cost. Energy density was expressed as kcal/100g. Nutrient density was based on the Nutrient Rich Food Index NRF_9.3_ (23). Retail food prices were obtained from local supermarkets Seattle-King County over the period of 12 years (2004–2016). The specific aims of the study were as follows—(a) To examine the quality of ultra-processed foods using a novel nutrient density metric, NRF_9.3_; (b) to examine the relative cost of NOVA categories, (c) to study trends in food prices over 12 years (2004–2016) by for unprocessed, processed and ultra-processed foods.

## Methods

The Fred Hutch (FHCRC) Food frequency questionnaire (FFQ) is a standard dietary data collection tool, widely used in large scale studies such as The Women' Health Initiative and Nurses' Health Study (4, 5).

The FHCRC FFQ was constructed based on 384 component or “recipe” foods that are commonly consumed in the US. The list of these foods was developed based on NHANES and Minnesota Nutrition database. The FFQ list of foods is very specific in terms of whether the item is fresh, frozen, or canned, and which are commercially available or prepared at home. Following those specifics, the lowest retail price at which each item was available were collected. Details of the FFQ methodology have been published (24).

### Classifying Foods by Food Groups and Processing

The present novel approach was to apply the NOVA classification scheme to the 384 component foods (Supplementary file) in the well-established Fred Hutch food frequency questionnaire (FFQ). First, each food was assigned into one of 7 USDA MyPyramid food groups: dairy; meat, poultry and fish; beans, nuts, and seeds; grains; fruit and juices; vegetables; and fats and sweets (25). Second, each food was also assigned into one of 4 NOVA categories: ultra-processed, processed, unprocessed and culinary ingredients (3). Two researchers applied the NOVA and MyPyramid food group classification independently on 384 FFQ food items. Coefficient correlation was applied to test the inter-rater reliability. There were only 8 items for which dis-concordance was found. Researchers then met to discuss those food items, and came to mutually agreed classification. Following published NOVA classification guidelines, unprocessed foods were defined as fruits, vegetables, grains, or meats that had been subjected to minimal or no processing. These could be fresh, dry, or frozen. Unprocessed foods included fresh meat, milk and plain yogurt, vegetables, eggs, legumes, fish, and other seafood, and unsalted nuts and seeds. Fruit juice was included if freshly squeezed. Tea and coffee were deemed to be unprocessed. Breads were unprocessed if simple and home-made.

Culinary ingredients were sugar, animal fats (butter) and vegetable oils, starches, salt, and vinegar (16). Processed foods were manufactured by adding culinary ingredients (fat, sugar, salt) to wholesome fresh foods. Those foods included cheese, ham, salted, smoked, or canned meat or fish, pickled vegetables, salted or sugared nuts, beer, and wine.

Ultra-processed foods were defined as industrial creations, which contained ingredients not found in home cooking, in addition to fat, sugar, and salt. Ultra-processed foods included commercial breads (refined and whole grain), ready-to-eat breakfast cereals, cakes, sweet snacks, and pizza, French fries, soft drinks (sodas and fruit drinks), ice cream, and frozen meals and soups. In the NOVA scheme, mass-produced whole grain breads, commercial sweetened yogurts, commercial fruit juices, and ready to eat cereals all fell into the ultra-processed category.

According to the NOVA classification, the most desirable foods were those that were fresh and minimally processed and were prepared, seasoned and cooked from scratch during ordinary culinary preparations at home (26).

### Developing Food Quality Metrics: Energy Density and NRF_9.3_

Energy density is the ratio of total energy intake over daily weight of total foods consumed (kcal/g) (27). Nutrient Rich Food Index 9.3 (NRF_9.3_) was used as a measure of nutrient density (23). The NRF_9.3_ assigns a nutrient quality score to each food item based on nine qualifying nutrients (protein, fiber, Vitamin A, C, and D, calcium, iron, magnesium, potassium) and three nutrients to limit (saturated fats, added sugar, and sodium) (23). The final score is the sum of percent daily values for 9 nutrients to encourage minus the sum of percent maximum recommended values for 3 nutrients to limit. All daily values were calculated per 100 kcal capped at 100% for positive nutrients.

(1) $$\begin{array}{l} NRF_{9.3}=\left(\sum_{1-9}\left(\text{Nutrient/DV}\right)\times 100\right)\\ -\left(\sum_{1-3}\left(\text{Nutrient/MRV}\right)\times 100\right) \end{array}$$

### The Seattle King County Food Prices Database: 2004–2016

Seattle's food prices database 2004–2016 contains lowest retail price for each of the FFQ component foods, collected in 3 large supermarket chains (Safeway, QFC, Albertsons) every 2 years from 2004 to 2016. Standardized data collection protocols, described in past studies, were used (24, 27). Each data collection period was between April and July to account for seasonality. Data were collected during in-store visits and compared to store website prices (Safeway) where available. Temporary promotions, specials, and discounts were excluded.

Shelf and unit prices were corrected for yield, using USDA Handbook 102 (28), to compute food prices per 100 g edible portion. Yield values reflect the edible proportion for each food item after taking losses due to inedible portions or cooking loss into account. Prices per 100 g were then adjusted for energy density for each food item to provide prices per 100 kcal. Price per 100 g of edible portion and price per 100 kcal served as the two primary indicators to study the cost gradient and time trends.

### Statistcial Analyses

Descriptive statistics examined the distribution of FHCRC food items by USDA food groups and by NOVA classification. Mean (SD) and median values of energy density and NRF9.3, and mean and median food prices ($/100 g and $/100 kcal) were computed for each group and processing category. ANOVA was applied to compare within group differences. Price trends were analyzed over the 12 years period (2004–2016). For analytical purpose, a list of 371 FFQ component foods were used after excluding 11 outliers (mostly fresh fish, fresh oysters, clams, halibut and crab) and 2 items with missing price data for one or more years. Sensitivity analyses were conducted before and after excluding the outliers. All statistical analyses were conducted using SPSS 22 statistical software and Microsoft Excel (2016).

## Results

**Figure 1:**
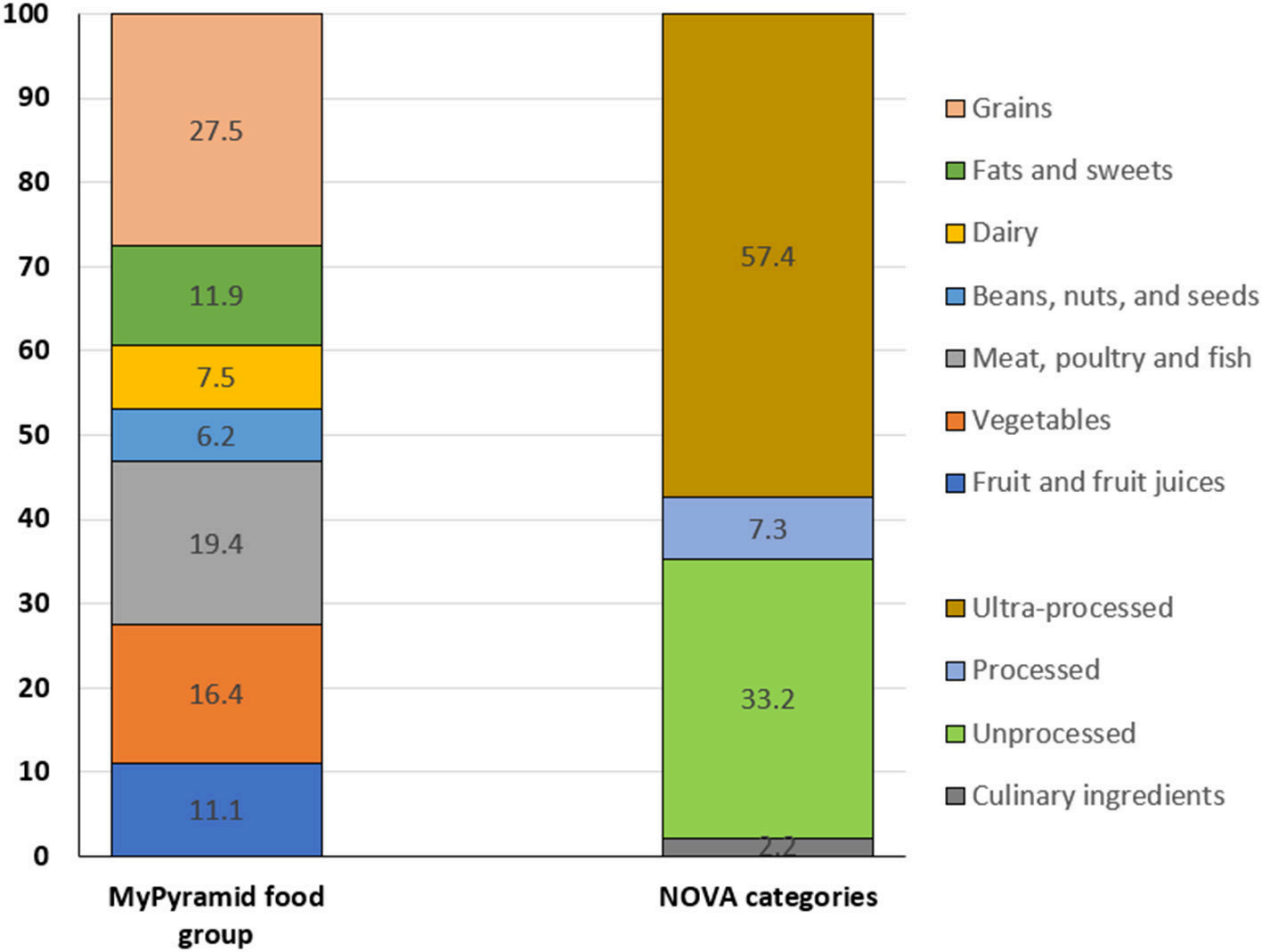
Percent distribution of FFQ component foods by USDA MyPyramid Food groups and NOVA categories.

Figure 1 shows the percent distribution of FFQ component foods by MyPyramid food groups and by 4 food processing categories. 27.5% of the FFQ foods were constituted by grains, followed by meat, poultry and fish (19.4%), vegetables (16.4%), fats and sweets (11.9%), fruits and fruit juices (11.1%), and 6.2% by beans, nuts, and seeds. More than half of the FFQ component foods (57%) fell into the ultra-processed category, with 33% into unprocessed category, and 7% in processed category.

**Figure 2:**
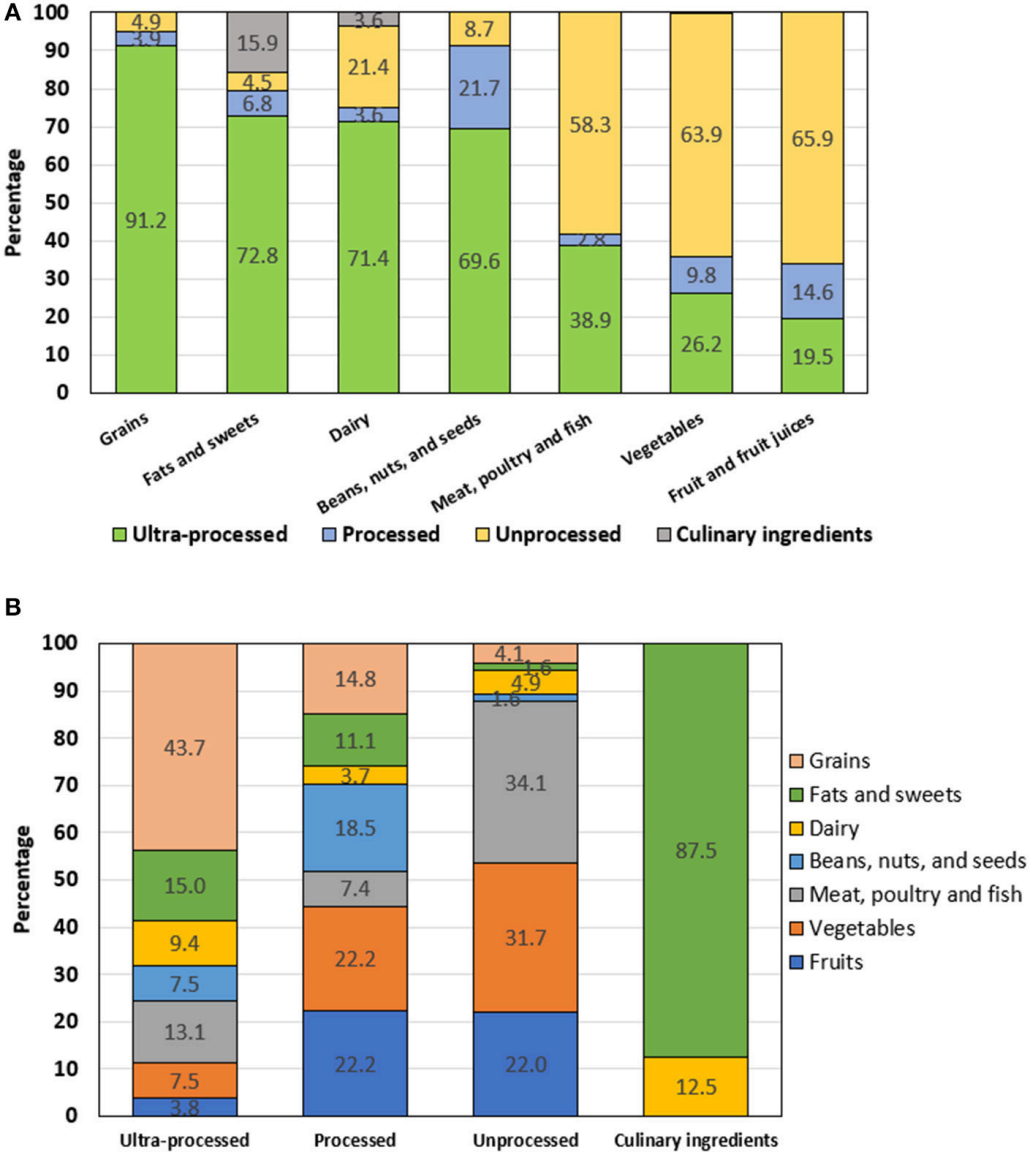
**(A)** Percent distribution of NOVA categories by MyPyramid food group. **(B)** Percent distribution of MyPyramid food groups by NOVA category.

Figure 2A shows the degree of food processing by each food group. Grains were pre-dominantly constituted by ultra-processed foods (91%), followed by fats and sweets (73%), dairy (71%), and beans, nuts and seeds (70%). Vegetables and fruits contained the lowest proportion of ultra-processed foods (26 and 20%, respectively). In other words, almost 60% of the vegetables, fruits and meat, poultry and fish groups were constituted by unprocessed foods in FHCRC FFQ. Figure 2B shows the reverse cross-tabulation, i.e., the distribution of each of 4 food processing categories by food groups. More than 40% of the ultra-processed foods were grains, followed by fats and sweets (15%), and meat, poultry, and fish (13.1%). Unprocessed foods, on the other hand, were mostly fruits and vegetables (54%), followed by meat, poultry, and fish (34%). Culinary ingredients were largely fats and sweets (87.5%).

Table 1 shows the gradient in food groups and food processing categories by two measures of quality (energy density and NRF 9.3 per 100 kcal). Among food groups, fruits, and vegetables had the highest NRF_9.3_ scores [mean (SD): 93.7 (67.3) and 150.0 (117.7), respectively] and the lowest energy density [0.6 (0.6) and 0.7 (0.7) kcal/g, respectively]. Fats and sweets had the highest energy density [2.9 (2.8) kcal/g] but lowest nutrient density [NRF: −1.08 (57.9)]. By degree of food processing, ultra-processed foods had higher energy density [2.3 (1.5) kcal/g] but lower NRF_9.3_ scores [21.2 (52.2)] than did unprocessed foods. Unprocessed foods had highest NRF_9.3_ score [108.5 (100.1)] and lowest energy density [1.10 (0.9) Kcal/g]. ANOVA was applied to compare within group difference. Both energy density and NRF values were statistically significant among both food groups and food processing categories.

**Table 1:** Energy density and nutrient density of 371 FFQ component foods by MyPyramid food group and NOVA category.

	**Energy density (Kcal/g)**				**Nutrient density (NRF 9.3) per 100 kcal**		
	***N***	**Mean (SD)**	**95% CI**	**Median (IQR)**	**Mean (SD)**	**95% CI**	**Median (IQR)**
**All items**	**371**	**1.96 (1.68)**	**[1.79, 2.13]**	**1.58 (2.22)**	**50.38 (81.95)**	**[41.99, 58.76]**	**21.40 (65.99)**
**MYPYRAMID FOOD GROUPS**							
Milk and milk products	28	1.66 (1.00)	[1.28, 2.05]	1.41 (1.12)	13.66 (29.67)	[2.16, 25.17]	4.18 (38.75)
Meat, poultry, and fish	72	2.01 (0.79)	[1.82, 2.19]	1.92 (0.86)	29.08 (36.32)	[20.55, 37.61]	23.41 (25.52)
Beans, nuts, and seeds	23	2.04 (1.91)	[1.21, 2.87]	1.13 (1.48)	46.73 (32.69)	[32.59, 60.86]	44.29 (53.26)
Grains	102	2.86 (1.38)	[2.59, 3.12]	2.89 (2.36)	21.39 (47.75)	[12.01, 30.77]	10.48 (25.79)
Vegetables	61	0.68 (0.69)	[0.51, 0.86]	0.37 (0.64)	150.01 (117.74)	[119.86, 180.16]	167.26 (201.07)
Fruits	41	0.66 (0.58)	[0.48, 0.84]	0.48 (0.25)	93.78 (67.30)	[72.26, 115.30]	86.70 (119.74)
Fats and sweets	44	2.93 (2.88)	[2.05, 3.80]	1.85 (4.35)	−1.08 (57.93)	[−18.91, 16.74]	−13.20 (48.10)
**NOVA CLASSIFICATION**							
Unprocessed	123	1.10 (0.87)	[0.95, 1.26]	0.84 (1.49)	108.50 (100.06)	[90.56, 126.43]	64.19 (144.64)
Processed	27	2.02 (2.03)	[1.22, 2.83]	0.85 (3.15)	37.95 (41.07)	[21.70, 54.19]	18.66 (48.84)
Ultra-processed	213	2.28 (1.54)	[2.07, 2.49]	2.00 (2.38)	21.23 (52.23)	[14.16, 28.30]	8.94 (36.56)
Culinary ingredients	8	6.36 (3.14)	[3.74, 8.99]	8.00 (5.59)	−21.78 (20.33)	[−38.78, −4.78]	−15.28 (40.32)

**Figure 3:**
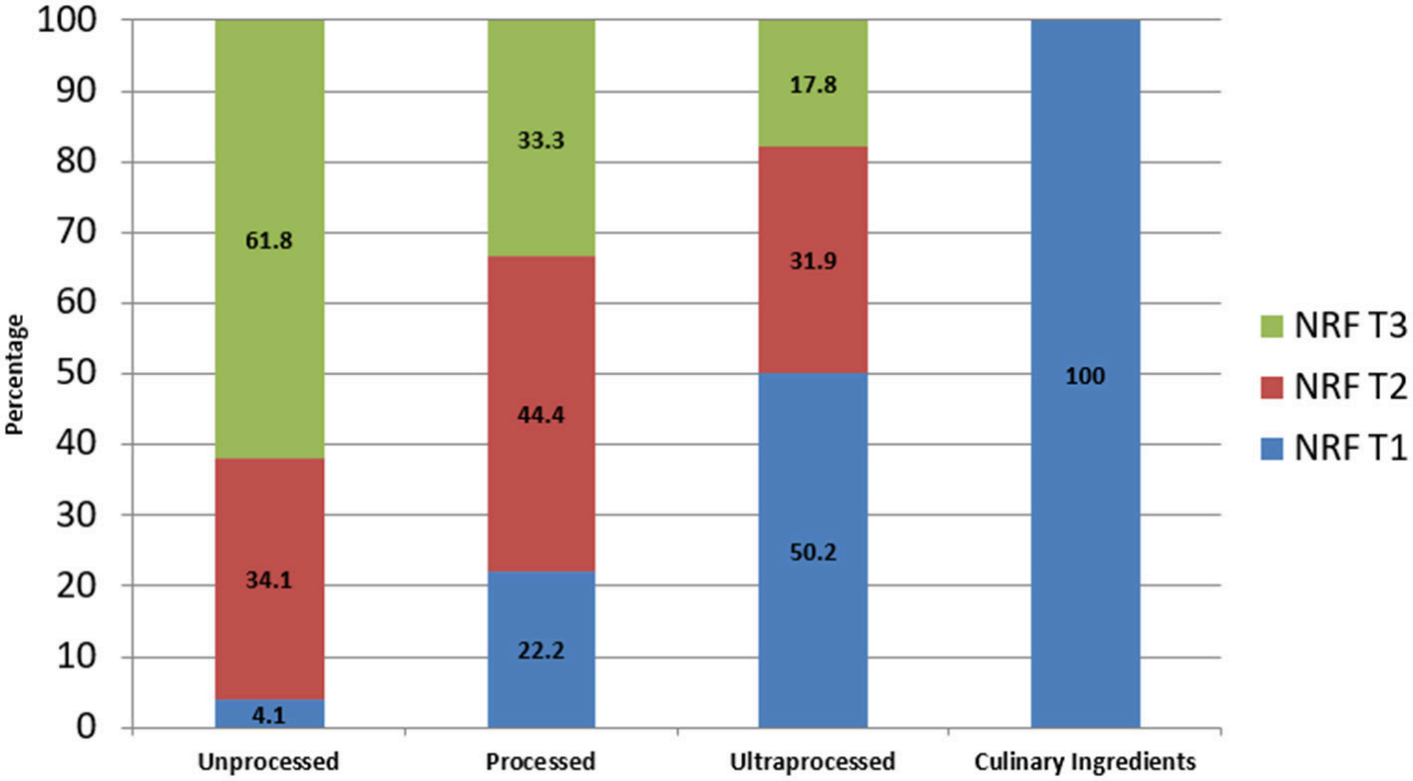
Percent distribution of NOVA categories by tertiles of NRF scores.

If we do tertile of NRF scores, 61% of unprocessed food fall into high NRF score category but only 4% in low NRF score category whereas 50% of ultra-processed food fall in low NRF score category and only 17% in high NRF score category (Figure 3). Unprocessed foods undoubtly fall in high NRF category as they have vitamins and mineral, have low energy density and are unprocessed/fresh like meat, fruit, and vegetable. However, some of the ultra-processed foods fall into nutrient rich category.

Table 2 shows the cost gradient, calculated per 100 kcal and per 100 g, for 371 FFQ component foods. Prices were compared across both food groups and food processing categories using ANOVA test. Vegetables and fruits had the highest cost per 100 kcal [mean (SD): 1.7 (1.4) and 1.3 (1.1), respectively], whereas grains and dairy were the lowest [mean (SD): 0.4 (0.4) and 0.4 (0.3), respectively]. Meat, poultry and fish, and nuts and seeds fell in the middle. By degree of food processing, ultra-processed foods cost $0.55/100 kcal; processed foods cost $0.64/100 kcal, and unprocessed foods cost $1.45/100 kcal. Prices for unprocessed foods were significantly above all other NOVA categories (*p* < 0.05). The lowest cost was for culinary ingredients, mostly fats, oils and sweeteners: $0.14/100 kcal.

**Table 2:** Mean 2016 food prices (USD per 100 g and 100 kcal edible portion) by food groups and food processing category.

	**Cost per 100 g**				**Cost per 100 kcal**		
	***N***	**$/100 g (SD)**	**95% CI**	**Median (IQR)**	**$/100 kcal (SD)**	**95% CI**	**Median (IQR)**
**All items**	**371**	**0.93 (0.76)**	**[0.86, 1.01]**	**0.72 (0.94)**	**0.85 (1.00)**	**[0.75, 0.95]**	**0.49 (0.80)**
**MYPYRAMID FOOD GROUPS**							
Grains	102	0.94 (0.60)	[0.82, 1.05]	0.89 (0.70)	0.39 (0.39)	[0.32, 0.47]	0.28 (0.30)
Fats and sweets	44	0.67 (0.75)	[0.44, 0.90]	0.44 (0.75)	0.64 (1.18)	[0.28, 1.00]	0.26 (0.47)
Milk and milk products	28	0.58 (0.62)	[0.34, 0.82]	0.37 (0.47)	0.37 (0.31)	[0.24, 0.48]	0.28 (0.24)
Beans, nuts, and seeds	23	0.90 (0.89)	[0.51, 1.28]	0.52 (0.72)	0.62 (0.61)	[0.36, 0.89]	0.39 (0.70)
Meat, poultry, and fish	72	1.58 (0.83)	[1.39, 1.78]	1.55 (1.07)	0.90 (0.59)	[0.76, 1.04]	0.81 (0.60)
Vegetables	61	0.66 (0.57)	[0.51, 0.80]	0.48 (0.43)	1.68 (1.44)	[1.31, 2.05]	1.25 (2.16)
Fruits	41	0.76 (0.60)	[0.57, 0.95]	0.58 (0.70)	1.33 (1.11)	[0.98, 1.68]	0.90 (1.48)
**NOVA CLASSIFICATION**							
Ultra-processed	213	0.93 (0.78)	[0.82, 1.04]	0.70 (0.88)	0.55 (0.61)	[0.47, 0.64]	0.38 (0.41)
Processed	27	0.71 (0.41)	[0.55, 0.87]	0.66 (0.60)	0.64 (0.48)	[0.45, 0.83]	0.53 (0.73)
Unprocessed	123	1.01 (0.79)	[0.86, 1.15]	0.76 (1.31)	1.45 (1.33)	[1.21, 1.68]	0.99 (1.52)
Culinary ingredients	8	0.73 (0.69)	[0.15, 1.30]	0.42 (0.82)	0.14 (0.12)	[0.04, 0.24]	0.10 (0.19)

**Figure 4:**
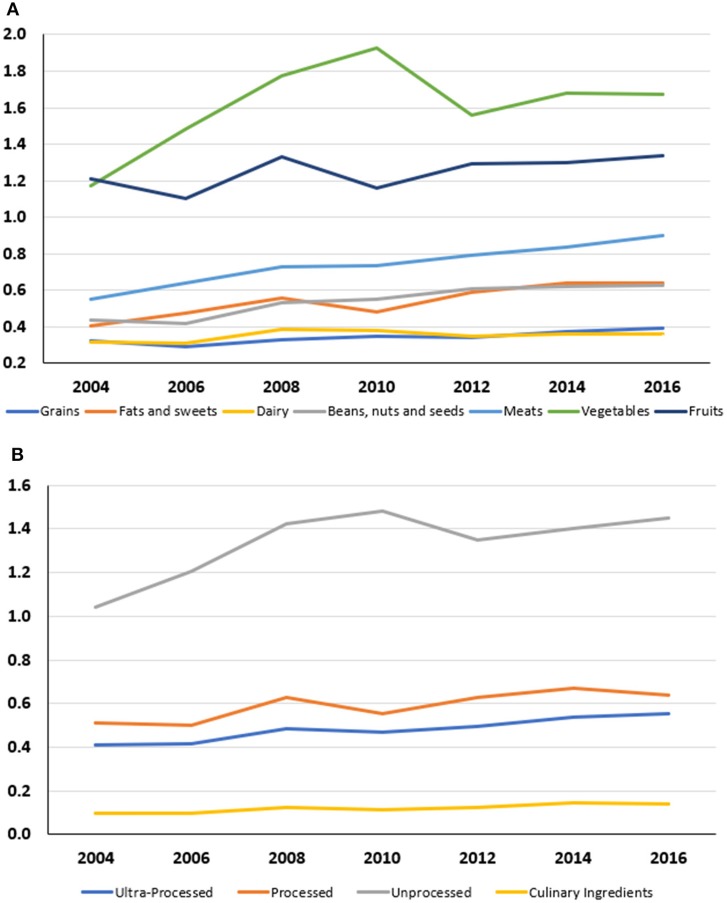
**(A)** Mean monetary cost in $/100 kcal for FFQ component foods by USDA MyPyramid Food groups (2004–2016). **(B)** Mean monetary cost in $/100 kcal for FFQ component foods by NOVA categories (2004–2016).

Mean food prices increased by about 37% from 2004 to 2016, as summarized in Figure 4. Ultra-processed foods (grains, fats and sweets) rose in price less than did unprocessed foods (fruit, vegetables and fresh meat, poultry and fish). On per calorie basis ($/100 kcal), price increases were $0.14 for ultra-processed foods $0.13 for processed food, and $0.41 for unprocessed foods, and 0.04$ for culinary ingredients.

## Discussion

This is one of the first few studies to explore nutrient density, energy density, and monetary cost of foods by the degree of processing. Ultra-processed foods were found to be low-cost, energy dense and nutrient poor as compared to unprocessed foods. These findings resonate with past studies suggesting ultra-processed foods as being energy-dense, high in saturated fat, added sugar, and salt and poor sources of protein, dietary fiber, and micronutrients (16, 29).

Utilizing component foods of a well-established FFQ instrument is one way to study the relative cost of different food groups. Separating the foods by NOVA classification and by the USDA food groups yielded some insights. First, as expected, most vegetables and fruits, fresh, frozen, or dried fell into the unprocessed category, as did meat, poultry and fish. Dairy was split into unprocessed (milk), and ultra-processed (commercial yogurt). Second, also as expected, unprocessed meat, poultry, fish, low fat milk, and vegetables and fruit had lower energy density and higher NRF_9.3_ nutrient density scores. Third, the unprocessed foods were more nutrient rich but they were also considerably more expensive.

The ultra-processed NOVA category captured not only fats and sweets (the stated intent) but also most of the commercially prepared breads and cereals, as well as beans, nuts, and seeds. Water, which provides weight but no calories, influences the energy density of foods more than does any macronutrient, including fat (30). As expected, ultra-processed grains, fats and sweets had higher energy density and lower NRF_9.3_ nutrient density scores than did unprocessed foods. Consistent with past observations (16, 17, 31), grains, fats and sweets cost less per calorie than did unprocessed foods.

It would appear that the ultra-processed NOVA designation is a new name for energy-dense grains, fats and sweets. These foods are energy dense, can be nutrient poor, and are distinguished by their low per calorie costs. By contrast, the NOVA unprocessed category successfully captured some of the same food groups that multiple NRF schemes have previously recognized as nutrient-rich.

The NOVA categories were characterized by sharply different food costs. Ultra-processed foods had lower NRF_9.3_ scores than did unprocessed foods but were also much less expensive. Dietary intake studies, applying the NOVA classifications to total diets, will determine how the NOVA classification is linked to sociodemographic determinants of diet choice: minority status, education, and incomes.

While Dietary Guidelines for Americans have emphasized the need to limit energy dense foods, and dietary sugars and fats, most of the US population is not meeting their nutrition goals (32). Having focused on nutrients to limit, the Dietary Guidelines have become more food oriented, specifying amounts of desirable foods and dietary ingredients in healthy food patterns. Food based dietary guidelines can take into account the nature of the food matrix which purely nutrient based calculations are unable to do. Reducing the share of ultra-processed foods in the diet has been suggested as an effective way to improve nutritional quality of diets (26).

The present study had limitations. First, it was based on a market basket of 371 FFQ foods and may not fully capture all the foods consumed by the US population. Second, the pricing was based on the lowest retail price for each item, and the same price was assigned across respondents. However, this is one of the standard widely-used procedures to study diet quality in relation to cost in the literature. The US Department of Agriculture calculates benefits for food assistance by attaching retail prices, similar to the ones here, to dietary intakes data.

Among the strengths of the study were the historical database collected every 2 years since 2004, utilizing the food database that builds the structure for one of the standard dietary data collection tools, and the potential to explore the cost of total diets featuring fresh vs. ultra-processed foods. The cost component was notably missing from virtually every study published on the topic of the NOVA classification scheme (2, 3, 16, 17, 26, 31, 33–36). Applying NOVA classification to dietary intakes, using standard dietary tools such as FFQ, will help placing processed and ultra-processed foods in the context of total diets.

## Conclusion

The study has implications for future research and policy efforts. Applying food processing classifications to prospective dietary studies will help clarify the impact of food processing on health outcomes. Food-based Dietary Guidelines focusing on dietary components or foods by degree of processing may be one strategy to make recommendations intuitive for the consumer; developing an overall processing index of the diet might be another.

## Data Availability

All datasets generated for this study are included in the manuscript and/or the Supplementary Files.

## Author Contributions

SG led data analyses. She assisted in conceptualizing the manuscript, data interpretation, and manuscript writing. TH led data collection and assisted in data analysis. AA led study data collection, assisted in conceptualizing the manuscript, data analyses, data interpretation, and manuscript writing. AD led the study design, data collection, conceptualization of the manuscript, and manuscript writing. All the authors reviewed and approved the submitted version of the manuscript.

### Conflict of Interest Statement

AD has received grants, honoraria, and consulting fees from numerous food, beverage, and ingredient companies and from other commercial and non-profit entities with an interest in nutrient density of individual foods, meals, and total diets. The remaining authors declare that the research was conducted in the absence of any commercial or financial relationships that could be construed as a potential conflict of interest.

## Abbreviations

FFQ: Food frequency questionnaires
NRF: Nutrient Rich Food
FHCRC: Fred Hutchinson Cancer Research Center
NHANES: National Health and Nutrition Examination Survey
RTE: Ready to Eat.
