# Supplementary material for: Characterizing Ultra-Processed Foods by Energy Density, Nutrient Density, and Cost
Source: Front Nutr. 2019 May 28;6:70. doi: 10.3389/fnut.2019.00070 (PMC6558394; doi:10.3389/fnut.2019.00070)
Supplement: Supplementary file 1 [file Data_Sheet_1.PDF]

FFQ Food Components

1 Margarine, diet
2 Margarine, stick
3 Margarine, tub
4 Butter
5 Lard
6 Oil, olive
7 Oil, canola
8 Oil, soybean/cottonseed
9 Sour cream
10 Tamale, with meat
11 Apples, fresh, with skin
12 Pear, fresh
13 pears, canned
14 Banana, fresh
15 Peaches, canned
16 Peaches, fresh
17 Nectarine, fresh
18 Plums, fresh
19 Apricots, fresh
20 apricots, canned
21 Apricots, dried
22 Prunes, dried
23 Raisins
24 Tangerines, fresh
25 Cantaloupe
26 Watermelon
27 Strawberries, fresh
28 kiwi, fresh
29 fruit cocktail
30 Blueberries, frozen
31 Applesauce
32 Grapes
33 pineapple, canned
34 orange jucie
35 Grapefruit juice
36 Kool-Aid
37 Hi-C
38 Grape juice
39 Apple juice
40 Mango, fresh
41 blueberries, fresh
42 blackberries, fresh
43 raspberries, fresh
44 pineapple, fresh
45 cherries, fresh
46 strawberries, frozen
47 Frutopia drink
48 Oranges, fresh
49 Grapefruit, fresh
50 String beans, canned
51 Green beans, fresh, cooked
52 Peas, canned
53 Peas, fresh, cooked
54 Beans, kidney, cooked
55 Beans, lima, dry
56 Beans, pinto, cooked
57 Corn, canned
58 Corn, fresh, cooked
59 Tomatoes, raw
60 Green pepper, raw
61 Peppers, red, raw
62 Broccoli, cooked
63 Spinach, cooked, from frozen
64 Collard greens, cooked
65 Carrots, raw
66 Carrots, cooked
67 Summer squash, cooked
68 Winter squash
69 Cauliflower, cooked
70 Cabbage, cooked
71 Sauerkraut
72 Brussel sprouts, cooked
73 Onions, cooked
74 Onions, green, raw
75 Salad dressing, Italian
76 French fries, fast food
77 Hashbrowns
78 Sweet potatoes, canned
79 Sweet potatoes, baked
80 Potato, baked, w/ skin

81 Potato, boiled, w/o skin
82 Potato, mashed w/ milk and fat
83 Salad, potato w/ mayo
84 Salad, macaroni w/ mayo
85 Rice, white
86 Rice, brown
87 Pasta, cooked
88 Dressing, salad, low calorie French
89 Dressing, salad, fat free Italian
90 spinach, fresh
91 lettuce, iceberg, fresh
92 lettuce, romaine, fresh
93 green peppers, cooked
94 peppers, jalapeno, raw
95 peppers, red, cooked
96 broccoli, raw
97 hominy
98 squash, zucchini
99 greens, mustard
100 onions, white, raw
101 garlic, cooked
102 avocado, fresh
103 guacamole
104 potatoes, fried
105 potatoes, mashed, dehydrated
106 beans, baked
107 chili, meatless
108 coleslaw, with mayo dress
109 juice, tomato
110 juice, v-8
111 ranch salad dressing, reg
112 cauliflower, raw
113 cabbage, raw
114 string beans, frozen,ckd
115 pumpkin, cnd
116 refried beans, cnd, reg
117 refried beans, cnd, fat free
118 refried beans, recipe
119 V-8 Splash
120 Beef, ground, regular
121 beef, chuck, arm, braised
122 beef, sirloin, wedge bone, broiled
123 pork, whole loin, roasted
124 Pork, ham, boneless, roasted, w/ fat
125 chili, canned with meat and beans
126 chili, homemade, beef and beans
127 Liver, beef
128 Liver, chicken
129 Organ meats, other
130 chicken, breast, fried w skin
131 chicken, breast, rstd w skin
132 chicken, breast, rstd w/o skin
133 chicken, thigh, rstd w/o skin
134 Sauce, white
135 Shrimp, fried
136 Clams, fried
137 Shrimp, not fried
138 crab
139 oysters
140 tuna, canned, oil, plain
141 Tuna salad w/ mayo, oil packed
142 tuna, canned, water, plain
143 Tuna salad,w/ mayo, water packed
144 Tuna casserole, water packed
145 sole, baked
146 salmon, baked
147 Bluefish
148 Macaroni and cheese
149 lasagna, homemade w meat sauce
150 Spaghetti with meat sauce, reg
151 Spaghetti sauce without meat
152 Chilaquiles
153 Quesadilla
154 Quesadilla with fat
155 enchilada, chicken
156 Tostada, bean and cheese
157 luncheon meat, ham
158 Bologna
159 Salami, cooked
160 Hotdog, regular

161 bratwurst, reg
162 Beef, ground, lean
163 Beef, ground, extra lean
164 Turkey, ground
165 Beef, chuck, trimmed
166 Beef, sirloin, trimmed
167 Pork, whole loin, trimmed
168 Ham, boneless, trimmed
169 Soup, tomato
170 Soup, cream of potato
171 Soup, clam chowder
172 Soup, bean with bacon
173 Soup, green pea
174 soup, lentil
175 Soup, vegetarian-vegetable
176 Soup, tortilla soup
177 Soup, tripe
178 Soup, chicken noodle
179 hot dog, lowfat
180 bratwurst, lowfat
181 luncheon meat, turkey
182 bologna, low fat
183 spam, cooked
184 lamb, roasted
185 meatloaf, ckd with reg grd beef
186 chicken, thigh, fried w skin
187 chicken nuggets
188 turkey, white and dark meat
189 oyster, fried
190 clams, not fried
191 lobster, not fried
192 snapper, baked
193 cod, baked
194 mackerel, baked
195 tofu, reg, firm
196 tofu, lowfat
197 tempeh
198 soy burgers
199 tofu hotdog
200 cheese, tofu
201 lamb, curried
202 chicken, pot pie
203 casserole, beef, mixed, with gravy
204 chicken, thigh, rstd w skin
205 casserole, chicken, mixed, creme sauce
206 fish fillets, cod, fried
207 fish fillet, commercial pre-breaded
208 pasta, alfredo sauce
209 pasta, with oil and parmesan
210 macaroni and cheese, boxed
211 rice, fried, pork
212 Chow mein, chicken
213 pad thai, vegetarian
214 pizza, meat, frozen
215 pizza, vegetable, frozen
216 burrito, bean, cheese
217 taco, beef, cheese
218 soup, minestrone
219 soup, cheese
220 soup, black bean
221 soup, miso
222 soup, ramen
223 fish, halibut, baked
224 stew, beef, homemade
225 turkey, white and dark, w/o skin
226 lamb, roasted, trimmed
227 lasagna, frozen
228 pizza, meat, fast food
229 pizza, vegetarian, fast food
230 enchilada, cheese
231 meatloaf, ckd with lean grd beef
232 meatloaf, ckd with x-lean grd beef
233 chicken, breast, grilled NFA w/o skin
234 chicken, thigh, grilled NFA w/o skin
235 chicken, breast, grilled w skin
236 chicken, thigh, grilled w skin
237 Oysters, Pacific, fried
238 Oysters, Pacific, not fried
239 Oysters, Eastern, Fried
240 Oysters, Eastern, not fried

241 granola, reg
242 Oatmeal
243 Grits, unknown, after cooking
244 pancake, plain, mix
245 waffle, homemade
246 Eggs, fried
247 Eggs, boiled
248 bacon
249 sausage, breakfast
250 Cereal, All Bran
251 Cereal, 40% bran
252 Cereal, Raisin Bran
253 Cereal, Cheerios
254 Shredded Wheat
255 Cereal, Total
256 Cereal, Product 19
257 Cereal, Cornflakes
258 cream of wheat, made w/water
259 oatmeal, flavored, pre-packaged, made w/water
260 french toast, plain
261 waffle, frozen
262 eggs, scrambled
263 Muffin, fruit
264 biscuit, plain
265 Bread, white
266 Bread, whole wheat
267 Cornbread, homemade
268 Cornbread, made from mix
269 Chips, potato
270 Chips, tortilla
271 Saltines
272 Popcorn, in oil
273 Peanuts, dry roasted
274 Peanut butter, smooth
275 mayo, real, reg
276 mayo, real, lowfat
277 mayo, real, nonfat
278 Popcorn, air-popped, no fat
279 scone, plain
280 croissant, plain
281 bagel, plain, white
282 bagel, plain, wheat
283 english muffin, plain, white
284 granola bar
285 cereal bar
286 power bar
287 tortilla chips, NF, Wow
288 pretzels
289 popcorn, microwave, LF
290 chips, potato, LF
291 popcorn, reg, microwave, buttered
292 snacks, cheese puffs
293 crackers, ritz
294 crackers, wheat thins
295 seeds, sunflower
296 nuts, mixed w/o peanuts
297 sauce cheese
298 gravy, turkey, made w/drippings
299 gravy, beef, made w/drippings
300 ketchup, reg
301 salsa, commercial
302 salsa, homemade
303 Clif bar
304 chips, corn
305 tortilla chips, lowfat, baked
306 chips, potato, NF, Wow
307 crackers, Ritz, reduced fat
308 crackers, Snackwells's, fat free
309 granola bar, lowfat
310 Tortilla, corn, plain
311 Tortilla, flour
312 Tortilla, flour, wheat
313 Cottage cheese, 2% fat
314 Cottage cheese, 4% fat
315 cheese, nonfat
316 cheese, mozzarella
317 Cheese, cheddar, reduced fat
318 Cheese, cheddar
319 Cheese, American, processed slices
320 Yogurt, nonfat, fruit

321 Yogurt, lowfat, plain
322 Milk, whole
323 Milk, 2%
324 Milk, 1%
325 Milk, skim
326 Cream
327 Cream, half and half
328 Cream, nondairy, liquid
329 Milk, soy, canned
330 cheese, ricotta, whole
331 cream cheese
332 Custard, baked
333 doughnut, raised
334 Cake, chocolate, frosted (20 oz)
335 cookies, oatmeal raisin
336 cookies, chocolate chip w/o nuts
337 neapolitan ice cream
338 Pie, apple, double crust (552g tot weight)
339 Pie, cherry, double crust(564 g tot weight)
340 Pie, chocolate cream (912 g)
341 Candy, milk chocolate, plain
342 Jelly, jam
343 Honey
344 cookies, figbars
345 gingersnap cookies
346 cookies, vanilla wafers
347 Cookies, graham cracker
348 Cake, angel food, unfrosted
349 doughnut, cake
350 pudding, choc, from a box with 2% milk
351 sherbet
352 cake, white, not frosted
353 cookies, choc chip, w/nuts
354 candy, snickers bar
355 candy, toffee
356 candy, lifesavers
357 icecream, choc, NF
358 ice cream, vanilla, Lf
359 frozen yogurt, NF, choc
360 frozen yogurt, LF, vanilla
361 shake, hard ice cream
362 shake, softserve
363 licorice
364 jelly beans
365 Soft drink, Cola
366 Soft drink, non-cola
367 Beer, regular
368 Wine, red
369 Wine, white
370 whiskey
371 Coffee, black
372 Tea, regular, black
373 Sugar, white
374 espresso
375 hot chocolate
376 slim-fast, liquid
377 ensure, liquid, cnd
378 instant breakfast, Carnation
379 soda, diet cola
380 soda, non-cola, diet
381 beve, water
382 Vitamin E
383 Vitamin C
384 Calcium
